# miR–483-5p promotes prostate cancer cell proliferation and invasion by targeting RBM5

**DOI:** 10.1590/S1677-5538.IBJU.2016.0595

**Published:** 2017

**Authors:** Zhi-Gang Yang, Xu-Dong Ma, Zhao-Hui He, Ying-xin Guo

**Affiliations:** 1 Department of Urology, Baotou Central Hospital, Inner Mongolia Medical University, China;; 2 Department of Urology, The First Affiliated Hospital of Guangzhou Medical University, China

**Keywords:** MIRN483 microRNA, human [Supplementary Concept], RBM5 protein, human [Supplementary Concept], Prostatic Neoplasms, Growth

## Abstract

**Objective:**

miR-483-5p has been identified as a miRNA oncogene in certain cancers. However, its role in prostate cancer has not been sufficiently investigated. In this study, we investigated the role of miR-483-5p in prostate cancer and examined RBM5 regulation by miR-483-5p.

**Material and methods:**

Expression levels of miR-483-5p were determined by quantitative real-time PCR. The effect of miR-483-5p on proliferation was evaluated by MTT assay, cell invasion was evaluated by trans-well invasion assays, and target protein expression was determined by western blotting in LNCaP, DU-145, and PC-3 cells. Luciferase reporter plasmids were constructed to confirm the action of miR-483-5p on downstream target gene RBM5 in HEK-293T cells.

**Results:**

we observed that miR-483-5p was upregulated in prostate cancer cell lines and tissues. A miR-483-5p inhibitor inhibited prostate cancer cell growth and invasion in DU-145 and PC-3 cells. miR-483-5p directly bound to the 3' untranslated region (3'UTR) of RBM5 in HEK-293T cells. RBM5 overexpression inhibited prostate cancer cell growth and invasion in LNCaP cells. Enforced RBM5 expression alleviated miR-483-5p promotion of prostate cancer cell growth and invasion in LNCaP cells.

**Conclusion:**

The present study describes a potential mechanism underlying a miR-483-5p/RBM5 link that contributes to prostate cancer development.

## INTRODUCTION

Prostate cancer is the most common type of cancer, and is an universal cause of cancer—related death in men worldwide (1). Therefore, it is necessary to improve prostate cancer detection, diagnosis, treatment and survival (2). However, there are few reliable biomarkers for early prostate cancer diagnosis and prognosis (3). Many microRNAs (miRNAs) have been shown to affect key cellular processes involved in prostate tumorigenesis, and thus, miRNAs may be potential prostate cancer biomarkers (4).

miRNAs are a group of small non-coding RNAs of 17-25 nucleotides in length that are conserved across species (5–7). miRNAs are involved in several developmental and physiological processes, and their dysregulation has been associated with disease development, including cancer (8, 9). They have been implicated in tumor formation, progression, invasion and metastasis. Depending on its target gene, a miRNA can act as an oncogene or tumor suppressor gene (10). Previous studies have suggested miR-483-5p as a potential hepatocellular carcinoma biomarker (11) and a marker of poor adrenocortical carcinoma prognosis (12, 13). Furthermore, miR-483-5p is a potential predictor of myeloma survival (14). It also promotes lung adenocarcinoma invasion and metastasis (15). miR-483-5p can be detected in the cell—free, non-exosome-enriched fraction of urine collected from patients with prostate cancer (16), however, its role in prostate cancer is unclear.

RBM5 is a well-known tumor suppressor gene, and it inhibits cell growth by modulating apoptosis (17). RBM5 inhibits lung adenocarcinoma formation through diverse apoptotic signaling pathways (18). RBM5 has been implicated as a tumor suppressor gene in lung cancer (19) and prostate cancer (20), but it is unclear whether RBM5 is a miR-483-5p target.

In this study, we explored the role of miR-483-5p in prostate cancer development. Our results suggested that miR-483-5p plays a critical role in cell proliferation and invasion by regulating its target gene RBM5 in human prostate cancer. The present study describes a potential mechanism underlying a miR-483-5p/RBM5 link that contributes to prostate cancer development. Our results demonstrated that miR-483-5p is a potential target in prostate cancer therapy.

## MATERIALS AND METHODS

### Cell lines

The human prostate cancer cell lines VCaP, LNCaP, DU-145, and PC-3, human prostate epithelial cell line RWPE-1, and HEK 293T cells were purchased from the American Type Culture Collection (ATCC). Prostate cancer cells were cultured in RPMI-1640 medium (Invitrogen) supplemented with 10% fetal bovine serum (Gibco) and in a 37°C humidified atmosphere of 5% CO_2_. RWPE-1 cells were cultured following the ATCC instructions. HEK 293T cells were grown in Dulbecco's modified Eagle's medium containing 10% fetal bovine serum (Gibco).

### Transfection

MiR-483-5p mimics and the miR-483-5p inhibitor were purchased from Sigma-Aldrich. We used mirVana miRNA mimic or mirVana miRNA inhibitor (Ambion, Austin, TX, USA) for the negative control. Furthermore, a RBM5 expression vector was generated into a pCMV-N-FLAG vector (Beyotime, Jiangsu, China) and pCMV-N-FLAG vector for the negative control. Cells were allowed to reach 70% to 80% confluence in 6-well plates before transfection. Cells were transfected using Lipofectamine^2000^ according to the manufacturer's instructions. After 48 hours of transfection, the cells were harvested for further study.

### Prostate tissues

Fresh tumor tissues were obtained from 26 prostate cancer patients during surgery at Baotou Central Hospital. The selected prostate cancer specimens were immediately frozen in liquid nitrogen and stored at −80°C for RNA extraction. The present study was approved by the Ethics Committee of Baotou Central Hospital.

### RNA preparation and quantitative real-time PCR

Total RNA was extracted from cells using TRIzol® Reagent (Invitrogen, Carlsbad, CA, USA) and treated with DNase I (Invitrogen, Carlsbad, CA, USA), according to the manufacturer's protocol. RNA (1μg) from each sample was reverse transcribed into complementary DNA (cDNA) using random primers, and the cDNA was subjected to quantitative reverse-transcription polymerase chain reaction (qRT-PCR). Subsequently, 1μg RNA was transcribed into cDNA using a miR-483-5p-specific stem-loop primer, and qRT-PCR was performed with miR-483-5p—specific primers using a 7500 Real-Time PCR System (Applied Biosystems, Mannheim, Germany). All miR-483-5p and U6 primers were synthesized by GenePharma, Shanghai.

### Protein extraction and Western blot analysis

Proteins were extracted with RIPA buffer (Beyotime, Shanghai, China) containing protease inhibitors. Equal amounts of protein samples were separated by 10% SDS-PAGE and electrotransferred to PVDF membranes (Millipore, Billerica, MA, USA). After blocking, the membranes were immunoblotted overnight at 4°C with primary antibodies, followed by HRP-conjugated secondary antibodies at 37°C for 1h. Signals were detected using an ECL system. Primary antibodies against RBM5 (Abcam) and GAPDH (KangChen Bio-tech) were used.

### Luciferase reporter assays

The RBM5 3'untranslated region (UTR) luciferase reporter plasmid and plasmid containing the RBM5 3'UTR (base pairs 416-438) with the miR-483-5p target site deleted were constructed using the pMIR-REPORT vector (Ambion, Austin, TX, USA). The two constructs were confirmed by DNA sequencing, and luciferase activity assays were performed. Briefly, luciferase activities were measured 48h post-transfection using a Dual-Luciferase Reporter Assay System (Promega, Beijing, China) following the manufacturer's instructions. The data were normalized by dividing firefly luciferase activity by that of Renilla luciferase.

### Cell proliferation assay

Cell proliferation was analyzed by 3- (4, 5-dimethylthiazol-2-yl)-2, 5-diphenyltetrazolium bromide (MTT) assay. Cells were seeded in 24-well plates and cultured for 1 to 4 days, following by the addition of MTT solution for 2 hours. After removing the medium, the remaining MTT formazan crystals were solubilized in DMSO and measured with a microplate reader at 570nm.

### Trans-well invasion assay

For invasion assays, 1.0×10^5^ cells were seeded in a Matrigel-coated chamber (BD Biosciences). Cells were seeded in serum-free media and then cultured in complete growth media. After 24 hours of incubation at 37°C, cells that had invaded were fixed and stained in dye solution containing 20% methanol and 0.1% crystal violet. Invasive cells were imaged using a BH-2 inverted microscope (Olympus). The mean values of three duplicate assays for each experimental condition were used for statistical analysis.

#### Statistical analysis

Statistical significance was determined using two-tailed Student's t-tests between the means of the control and experimental groups. All statistical calculations were performed and graphs were generated using Graphpad Prism 5.0 software.

## RESULTS

### miR-483-5p is significantly upregulated in prostate cancer cell lines and tissues

**Figure 1:**
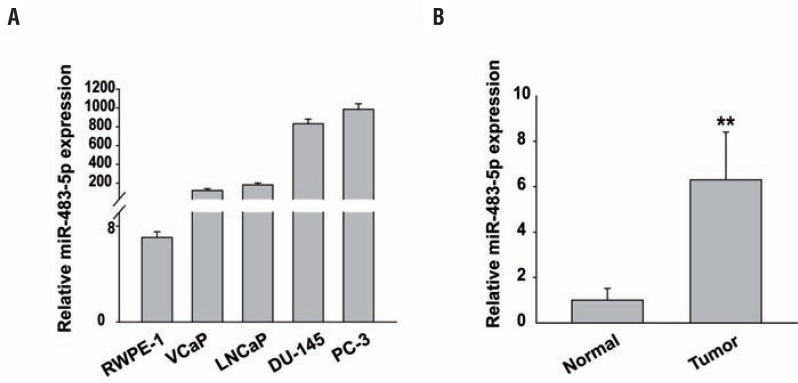
miR-483-5p is significantly upregulated in prostate cancer cell lines and tissues. A, histograms of the average relative expression of miR-483-5p in a normal prostate epithelial cell line and prostate cancer cell lines as shown. B, relative ratios of miR-483-5p expression in 26 prostate cancer tissues compared with 10 normal prostate tissues. **, P<0.01. Data are presented as mean±SD from three independent experiments.

We first performed quantitative PCR to detect miR-483-5p levels in the following prostate cancer cell lines: VCaP, LNCaP, DU-145, and PC-3. We compared them to the miR-483-5p levels in the non-tumorigenic RWPE-1 human prostate epithelial cell line. miR-483-5p was upregulated in all 4 prostate cancer cell lines compared to RWPE-1 cells (Figure-1A). To ascertain the clinical significance of miR-483-5p, we analyzed miR-483-5p expression levels by qRT-PCR in human prostate cancer tissues compared with its expression in normal human prostate tissues. We found that miR-483-5p expression dramatically increased in prostate cancer tissues (P<0.01, Figure-1B).

### MiR-483-5p inhibition decreases prostate cancer cell growth and invasion

**Figure 2:**
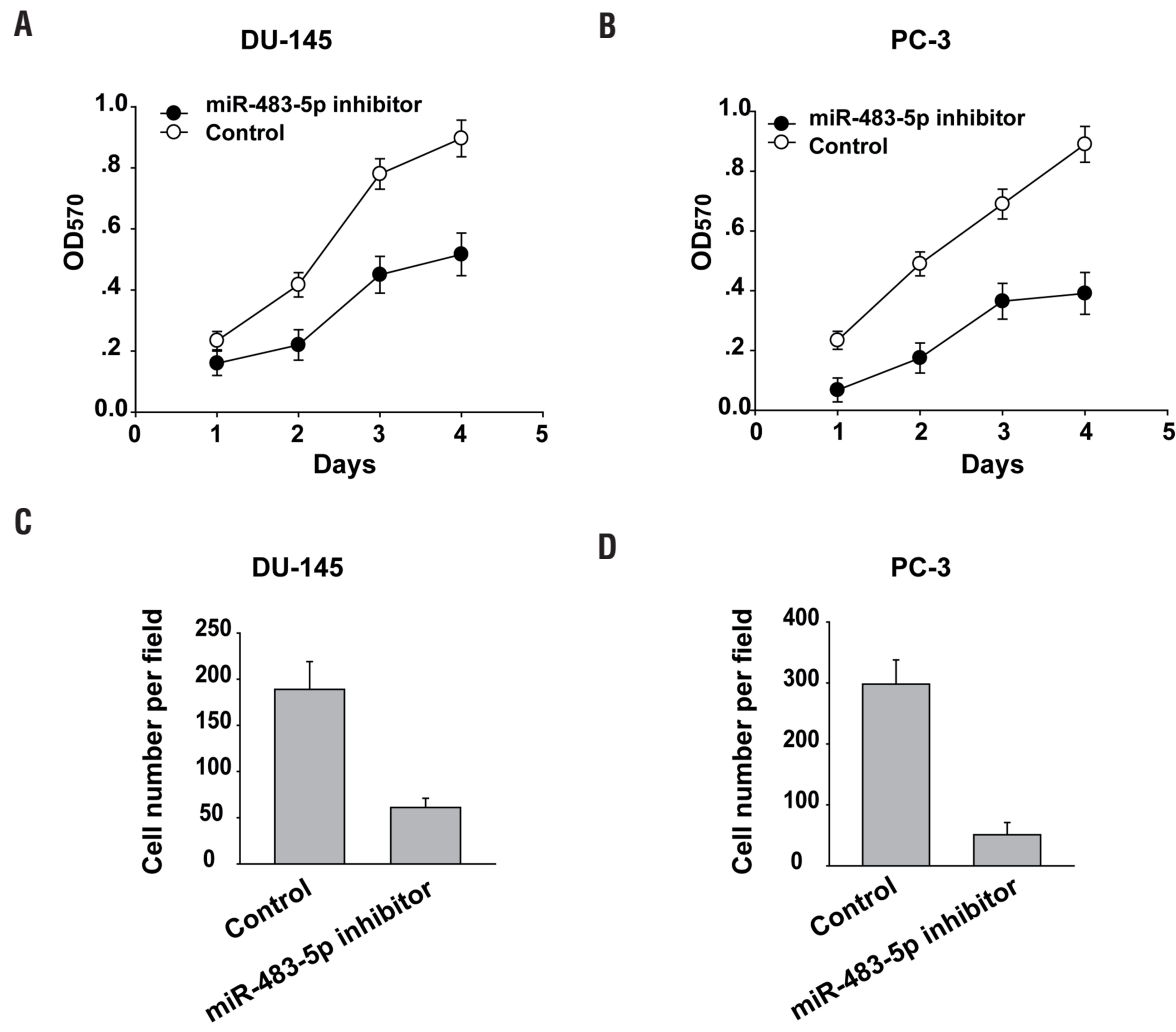
MiR-483-5p inhibition decreases prostate cancer cell growth and invasion. (A) (B) MTT assay was performed after transfection of Du-145 cells (A) or pc-3 cells (B) with the miR-483-5p inhibitor for the indicated time. (c) (D) Invasion assays were used to determine Du-145 cells (c) or pc-3 cells (D) motility. Experiments were performed in triplicate. Data are presented as mean±SD from three independent experiments.

To determine whether miR-483-5p promotes oncogenic phenotypes of prostate cancer, we performed inhibition function assays in prostate cancer cells by using a miR-483-5p inhibitor. As shown in Figures 2A and 2B, miR-483-5p inhibition significantly reduced prostate cancer cell growth compared to the scramble control cells as measured by MTT assay. Furthermore, we performed Trans-well invasion assays to determine whether miR-483-5p regulates prostate cancer cell invasiveness (Figures 2C and 2D). We found that miR-483-5p inhibition reduced prostate cancer cell invasion through Matrigel.

### RBM5 is a direct miR-483-5p target gene in prostate cancer

**Figure 3:**
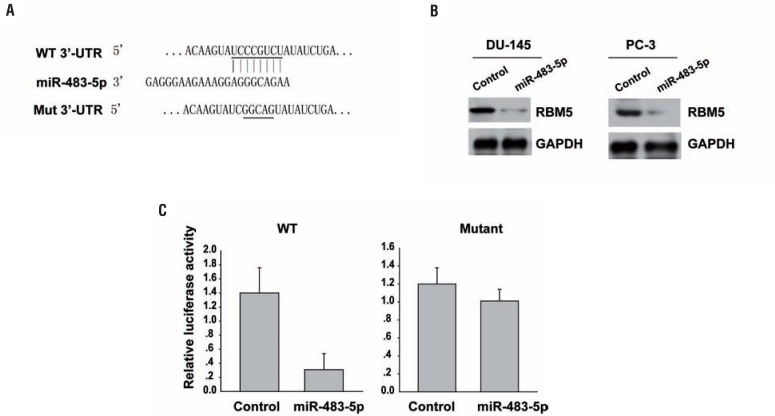
RBM5 is a direct miR-483-5p target gene in prostate cancer. (A) schematic representation of human RBM5 mRnA; highly conserved miR-483-5p binding sites were located in the 3' UTR. (B) RBM5 levels were examined by western blot analysis. GAPDH was also determined as a loading control. (c) Luciferase assay results from HEK-293T cells co-transfected with the RBM5 3' UTR-wt or RBM5 3' UTR-mut reporter plasmids and either miR-483-5p or miR-NC. Data are presented as mean±SD from three independent experiments.

By utilizing a bioinformatic algorithm (miRNAda), we identified RBM5 as a miR-483-5p target gene. The predicted miR-483-5p binding site within the RBM5 mRNA 3' UTR is shown in Figure-3A. Western blot analysis further demonstrated that miR-483-5p overexpression dramatically suppressed endogenous RBM5 protein levels (Figure-3B).

We demonstrated that miR-483-5p directly regulates RBM5 using a Dual-Luciferase Reporter Assay System. miR-483-5p strongly reduced luciferase activity only in the presence of the RBM5 3' UTR. Mutation of the miR-483-5p seed recognition motif abrogated these effects, confirming that RBM5 is a miR-483-5p target (Figure-3C).

### Enforced RBM5 expression mitigates miR-483-5p promotion of prostate cancer cell growth and invasion

**Figure 4:**
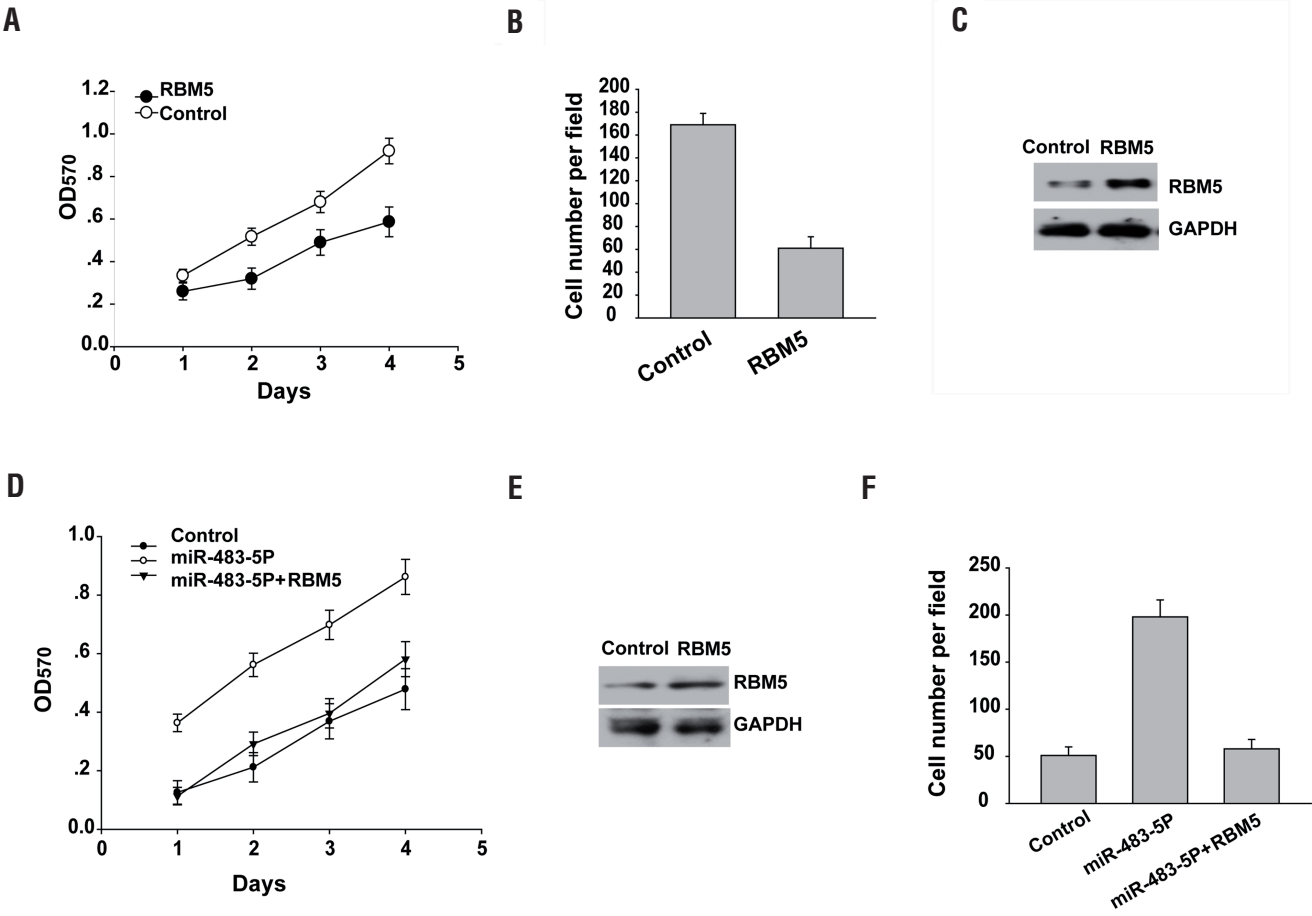
Enforced RBM5 expression mitigates miR-483-5p promotion of prostate cancer cell growth and invasion. (A) MTT assay was performed after transfection of LNCaP cells for 1 to 4 days. (B) Invasion assays were used to determine LNCaP cell motility. The invaded cell numbers were quantified 24h after cells were seeded. Data are presented as mean±sD from three independent experiments. (c) western blot analysis of RBM5 levels in the fourth day was examined. GAPDH was also determined as a loading control. (D) MTT assay was performed after transfection of Lncap cells for 1 to 4 days. (E) western blot analysis of RBM5 levels in the fourth day was examined. GAPDH was also determined as a loading control. (f) Invasion assays were used to determine LNCaP cell motility. The invaded cell numbers were quantified 24h after cells were seeded. Data are presented as mean±SD from three independent experiments.

We next investigated the role of RBM5 in prostate cancer progression. RBM5 overexpression reduced cell growth (Figure-4A) by MTT assay and invasion by trans-well invasion assay (Figure-4B), respectively. RBM5 overexpression was confirmed by Western blot analysis (Figure-4C). There results suggested that RBM5 inhibits prostate cancer cell growth and invasion.

To determine whether miR-483-5p targeting of RBM5 was required for inhibiting prostate cancer cell proliferation and invasion, we employed an expression construct that encodes the entire RBM5 coding sequence but lacks the 3′-UTR. Enforced RBM5 expression partially rescued the miR-483—5p-mediated decrease in cell growth by MTT assay and invasion by trans-well invasion assay (Figures 4D and 4F), respectively. RBM5 overexpression was confirmed by Western blot analysis (Figure-4E).

## DISCUSSION

Prostate cancer is one of the leading causes of cancer-related mortality. Although major breakthroughs in prostate cancer treatment have been achieved in recent years, it is important to identify novel and versatile molecular biomarkers for the disease (21).

Emerging evidence suggest that the dysregulation of miRNAs contributes to carcinogenesis. Moreover, accumulating evidence has demonstrated miR-483-5p expression in multiple types of tumors, including tongue squamous cell carcinoma, multiple myeloma, lung adenocarcinoma, hepatocellular carcinoma, and glioma (11–15, 22, 23). However, it remains unclear whether miR-483-5p is expressed in prostate cancer and whether it plays a role in prostate cancer.

In the present study, we observed increased miR-483-5p expression in prostate cancer cell lines or tissues compared to a normal prostate epithelial cell line tissues. Moreover, miR-483-5p inhibition suppressed prostate cancer cell growth and invasion in vitro. In the future, we will further perform analysis of miRNA arrays to reveal many genes difference in expression between benign and malignant tissue samples.

Despite previous studies indicating the oncogenic role of miR-483-5p, its role in tumor cell growth and invasion and its molecular mechanisms regulating growth and invasion are unknown. Here, we found that miR-483-5p expression was upregulated in prostate cancer cell lines. Moreover, we identified miR-483-5p as a pro-metastatic miRNA and a negative regulator of the key metastasis suppressor RBM5. Our study describes the regulatory link between miR-483-5p and RBM5 and identifies a potential mechanism of RBM5 dysregulation and its contribution to prostate cancer progression. We found that miR-483-5p expression is significantly upregulated in prostate cancer cell lines and is negatively associated with RBM5 protein levels. miR-483-5p suppression inhibits prostate cancer cell growth and invasion by directly targeting RBM5. However, further investigation in other types of cancer is necessary to explore the function and mechanisms of miR-483-5p.

In summary, the present study is the first to suggest the potential prognostic significance of miR-483-5p mediated the downregulation of RBM5 expression that promotes cancer cell proliferation and invasion in prostate cancer.
